# Monitoring of Insecticide Resistance and Resistance-Related Point Mutations in Field-Collected *Aphis gossypii* Populations in the Northern Xinjiang, China

**DOI:** 10.3390/insects17030314

**Published:** 2026-03-13

**Authors:** Yunhao Wang, Wenjie Li, Mei Liu, Renci Xiong, Yongsheng Yao, Wei Wang

**Affiliations:** 1 College of Agriculture, Tarim University, Aral 843300, China; wyh121209@163.com (Y.W.); liwenjie010719@163.com (W.L.); zlmo829@163.com (M.L.); xiongrc@taru.edu.cn (R.X.); 2 Key Laboratory of Integrated Pest Management on Crops in Northwestern Oasis, National Plant Protection Scientific Observation and Experiment Station of Korla, Institute of Plant Protection, Ministry of Agriculture and Rural Affairs, Xinjiang Uygur Autonomous Region Academy of Agricultural Sciences, Urumqi 830091, China

**Keywords:** *Aphis gossypii*, resistance monitoring, gene mutation

## Abstract

In 2024 and 2025, field populations of *Aphis gossypii* were collected from eight regions in Xinjiang to monitor their resistance levels to five commonly used insecticides: sulfoxaflor, acetamiprid, imidacloprid, abamectin, and chlorpyrifos. The mutation frequencies of five sites in the acetylcholinesterase (AChE) gene (S431F, V332A, A302S, G221A, F139L) and three sites in the *β*1 subunit of the nicotinic acetylcholine receptor (nAChR) (R81T, V62I, K264E) were also analyzed. The results showed that from 2024 to 2025, the eight *A. gossypii* field populations exhibited the highest resistance to imidacloprid (primarily moderate to high resistance), followed by acetamiprid (all moderate resistance). Resistance to abamectin and sulfoxaflor was relatively low, but sulfoxaflor resistance increased rapidly (from low resistance in 2024 to moderate resistance in 2025). All populations remained consistently susceptible to chlorpyrifos. Gene analysis revealed that the mutation rate of S431F in the AChE gene was nearly 100%, while that of V332A remained stable at approximately 30%. The mutation rates of A302S and G221A showed a slight increase, whereas the F139L mutation rate was extremely low (<1.00%). In the *β*1 subunit of nAChR, the mutation rates of R81T and V62I remained stable at around 50%, and the K264E mutation rate was extremely low (<1.00%). This study clarifies the resistance evolution patterns of *A. gossypii* to different insecticides and the variation characteristics of key resistance genes in Xinjiang, providing a scientific basis for the integrated resistance management of *A. gossypii* and the rational selection of effective insecticides.

## Simple Summary

Between 2024 and 2025, we collected cotton aphids (*Aphis gossypii*) from eight regions in Xinjiang to evaluate their resistance levels to five commonly used insecticides and to monitor genetic mutations associated with such resistance. Our findings indicate that these aphid population exhibited the strongest resistance to imidacloprid, mostly at moderate-to-high levels, followed by moderate resistance to acetamiprid across all sampled populations. Resistance to abamectin and sulfoxaflor remained relatively low, but sulfoxaflor resistance increased from low to moderate levels within a single year. All aphid groups maintained high susceptibility to chlorpyrifos. Regarding the target-site mutations examined, one genetic locus demonstrated a near-complete mutation frequency (100%), another remained stable at approximately 30%, two showed minor increases, while others maintained minimal mutation frequencies. This study elucidates the evolutionary patterns of insecticide resistance in Xinjiang’s cotton aphid populations and documents corresponding genetic changes, providing valuable insights for agricultural practitioners to optimize insecticide selection and implement effective resistance management strategies for cotton crop protection.

## 1. Introduction

The cotton aphid, *Aphis gossypii* Glover, is a major pest that poses a considerable threat to cotton production in Xinjiang [1,2]. It frequently aggregates on the tender parts of cotton plants or the undersides of new leaves, feeding on sap with its piercing–sucking mouthparts [3]. This feeding behavior causes leaf curling, stunted growth, and the shedding of flower buds and bolls, thereby affecting normal cotton development [4,5,6]. Meanwhile, the honeydew secreted by *A. gossypii* adheres to leaves, hindering photosynthesis and respiration while promoting fungal growth [7], ultimately reducing cotton quality and yield.

Although agricultural, physical, and biological control methods are important components of the integrated pest management system for *A. gossypii* in cotton fields, chemical control remains the primary measure for suppressing aphid populations and mitigating immediate damage [8,9]. However, long-term and excessive reliance on a limited range of insecticides has imposed strong selection pressure [10], accelerating the evolution of resistance in *A. gossypii* and diminishing the efficacy of conventional insecticides. Field monitoring data from Yining, Bole, Kuitun, Shihezi, Wujiaqu and Korla in Xinjiang revealed that *A. gossypii* populations exhibited resistance ratios of 85.20–412.00-fold, 221.00–777.00-fold, and 122.00–1095.00-fold to imidacloprid, acetamiprid, and thiamethoxam, respectively, in 2018, all reaching moderate-to-high resistance levels [11]. By 2020, *A. gossypii* in Bole, Changji, Kuytun, Shawan, Shihezi, Wusu, and Yining in northern Xinjiang demonstrated extremely high resistance to imidacloprid, with resistance ratios ranging from 3516.10- to 31,186.46-fold, while populations in Alar, Kashgar, and Korla in southern Xinjiang showed resistance ratios of 174.70- to 2215.94-fold [12]. Beyond neonicotinoids, the sulfoximine insecticide sulfoxaflor has also demonstrated declining efficacy against *A. gossypii* in Xinjiang. In 2017, *A. gossypii* populations in Kuitun, Wujiaqu, Shihezi, Korla, and Hami were susceptible to sulfoxaflor, with resistance ratios ranging from 1.43- to 3.65-fold [13]. However, by 2020, resistance ratios to sulfoxaflor had increased to 13.50–30.08-fold in northern Xinjiang (Bole, Changji, Kuitun, Shawan, Shihezi, Wusu, Yining) and 7.95–22.80-fold in southern Xinjiang (Alar, Kashgar, Korla) [12].

The primary molecular mechanisms of insecticide resistance in *A. gossypii* include metabolic resistance and target-site resistance [14,15]. *A. gossypii* has developed resistance to multiple insecticides, including acetamiprid, thiamethoxam, imidacloprid, and sulfoxaflor, by enhancing the detoxification and metabolic capabilities of cytochrome P450 monooxygenases (P450s), carboxylesterases (CarEs), glutathione S-transferases (GSTs), ATP-binding cassette transporters (ABCs), and UDP-glycosyltransferases (UGTs) [16,17,18,19,20,21]. Metabolic resistance mechanisms also play a significant role in other hemipteran pests; for example, elevated activities of detoxification enzymes have been demonstrated to contribute substantially to organophosphate resistance in field populations of the whitefly *Bemisia tabaci* [22]. In addition to these well-established mechanisms, emerging evidence suggests that intestinal commensal bacteria may also play an auxiliary role in resistance development. This effect may be achieved through direct metabolism of insecticides or modulation of host detoxification pathways [23]. For instance, *Sphingomonas* sp. has been shown to metabolize imidacloprid, thereby potentially enhancing resistance of *A. gossypii* to this compound, although the level of symbiont-mediated resistance is typically low [24]. Furthermore, alterations in the structure or expression levels of target proteins represent another important mechanism of resistance in *A. gossypii* [14]. Mutations in the acetylcholinesterase gene can affect the susceptibility of *A. gossypii* to carbamate and organophosphate insecticides—for example, the S431F and A302S mutations are associated with resistance to these insecticides [25]. Mutations in the nicotinic acetylcholine receptor *β*1 subunit (such as L80S, R81T, V62I, and K264E) are closely linked to neonicotinoid resistance in *A. gossypii* [26,27]. Similarly, mutations in the voltage-gated sodium channel gene (L1014F and M918L/V) can confer high levels of resistance to pyrethroid insecticides in *A. gossypii* [28,29].

Cotton is a significant cash crop in Xinjiang, and its production security directly influences regional agricultural economic stability and farmer income. Nevertheless, increasing insecticide resistance in *A. gossypii* has become a critical constraint on the green and high-quality development of Xinjiang’s cotton industry. Therefore, accurately assessing the insecticide resistance levels of *A. gossypii* across different cotton-growing regions in Xinjiang and characterizing the mutation s in resistance-associated genes are essential prerequisites for developing scientifically sound control strategies. In this study, we monitored the insecticide resistance of field populations of *A. gossypii* and systematically examined mutation frequencies of five sites in the AChE gene (A302S, V332A, S431F, F139L, G221A) and three sites in the nAChR *β*1 subunit (R81T, K264E, V62I). The objective was to clarify the current status and evolutionary trends of insecticide resistance in *A. gossypii* in Xinjiang, thereby providing a scientific foundation for precise pest control and resistance management.

## 2. Materials and Methods

### 2.1. Insect

The field populations of the tested *A. gossypii* were collected separately from cotton-growing areas in northern Xinjiang during July 2024 and 2025, with samples obtained from seven counties and cities each year. The collection sites in 2024 included Hutubi (HTB, 44°10′28″ N, 86°35′52″ E), Manas (MNS, 44°12′48″ N, 86°22′5″ E), Shihezi (SHZ, 44°17′12″ N, 85°57′29″ E), Shawan (SW, 44°18′45″ N, 85°42′6″ E), Wusu (WS, 44°22′21″ N, 84°18′36″ E), Jinghe (JH, 44°35′15″ N, 82°54′7″ E) and Bole (BL, 44°46′54″ N, 82°20′3″ E), while those in 2025 comprised Hutubi (HTB, 44°8′49″ N, 86°52′36″ E), Manas (MNS, 44°11′20″ N, 86°29′36″ E), Shihezi (SHZ, 44°17′16″ N, 85°57′37″ E), Shawan (SW, 44°17′18″ N, 85°49′34″ E), Wusu (WS, 44°27′31″ N, 84°40′43″ E), Jinghe (JH, 44°35′7″ N, 82°54′9″ E) and Kuitun (KT, 44°25′12″ N, 84°58′0″ E). The sampling sites are detailed in Table 1. All collected aphid samples were identified under a stereomicroscope based on morphological characteristics, with key features including black cylindrical siphunculi and finger-shaped cauda with 2–3 setae on each side, confirming the species as *A. gossypii* Glover. The susceptible population of *A. gossypii* used in this study was originally collected from a field population in Jinghe County, Xinjiang, in July 2019. These specimens were subsequently reared in the laboratory for over 50 generations without exposure to insecticides. All populations were maintained in a climate chamber under controlled conditions: temperature 26 ± 1 °C, relative humidity 70 ± 10%, and a photoperiod of 16 L:8 D.

### 2.2. Insecticides and Reagents

Sulfoxaflor (95% *w*/*w*, sulfoximine insecticide) was obtained from Corteva Agriscience (Indianapolis, IN, USA). Acetamiprid (97% *w*/*w*, neonicotinoid insecticide), chlorpyrifos (97% *w*/*w*, organophosphate insecticide), abamectin (96% *w*/*w*, macrocyclic lactone insecticide), and imidacloprid (neonicotinoid insecticide) were obtained from Jiangsu Weier Chemical Co., Ltd. (Yancheng, China). Triton X-100 was obtained from Beijing Coolaber Technology Co., Ltd. (Beijing, China). All other chemicals and solvents were analytical-grade reagents obtained from Sinopharm Chemical Reagent Co., Ltd. (Shanghai, China).

### 2.3. Bioassays

The leaf-dipping method was adopted to assess the toxicity of sulfoxaflor, acetamiprid, imidacloprid, abamectin, and chlorpyrifos against field population of *A. gossypii*. The tested insecticides were prepared by dissolving them in N,N-dimethylformamide at a concentration of 10,000 mg/L. These stock solutions were subsequently diluted into five concentration gradients using 0.05% (*v*/*v*) Triton X-100 solution. A 0.05% (*v*/*v*) Triton X-100 solution served as the blank control. Fresh cotton leaves were carefully punched into circular leaf disks with a diameter of 23 mm. The discs were then immersed in the insecticide solutions for 15 s and allowed to air-dry naturally in a cool, shaded area. The dried leaf disks were placed upside-down on 1.5% agar beds (2 mL per well) in 12-well cell culture plates. Each leaf disk was inoculated with 30 apterous adult aphids, and the plates were sealed with rice paper to prevent aphid escape. The inoculated plates were maintained in an artificial climate chamber under controlled conditions: temperature (26 ± 1) °C, relative humidity (70 ± 10)%, and a photoperiod of 16:8 L/D for rearing. Mortality was recoded after 48 h of exposure, with three biological replicates per concentration gradient.

### 2.4. Detection of Gene Mutation Frequency

Amplicon sequencing was used to determine the mutation frequencies of three point mutations (R81T, K264E and V62I) in the *β*1 subunit of the nicotinic acetylcholine receptor (nAChR), as well as five point mutations (A302S, V332A, S431F, G221A and F139L) in the acetylcholinesterase (AChE) gene of *A. gossypii*.

The sequences used in this study are based on the following GenBank accession numbers: AChE1: XP_027848419.1; AChE2: XP_027850887.2; nAChR *β*1: XP_027842152.1. Mutation nomenclature combines the amino acid position with the specific substitution; for example, the R81T mutation in nAChR *β*1 indicates that the original arginine (R) at position 81 is substituted with threonine (T).

To ensure that the target mutation sites were located in the central region of the amplified sequences, specific upstream and downstream primers were designed using Primer3Plus (https://www.primer3plus.com/, accessed on 31 October 2024) (Table S1), with the goal of amplifying the target gene fragments to approximately 250 bp in length. All primers were synthesized by Sangon Biotech (Shanghai) Co., Ltd., Shanghai, China. A total of 100 adult aphids were randomly selected from each field population and pooled in the same centrifuge tube. The genomic DNA from each population was extracted separately using the FastPure Cell/Tissue DNA Isolation Mini Kit-BOX 1 (Vazyme Biotech Co., Ltd., Nanjing, China). The extracted genomic DNA was then used as a template for PCR amplification, performed with the aforementioned specific primers and 2 × Rapid Taq Master Mix (Vazyme Biotech Co., Ltd., Nanjing, China). After amplification, agarose gel electrophoresis was performed on the PCR products to confirm the size of the target bands. All PCR products were subsequently submitted to Ascend Biotechnology (Nanjing) Co., Ltd., Nanjing, China. for further processing, including library preparation, sequencing, and amino acid mutation detection.

### 2.5. Statistical Analyses

The experimental data were analyzed using the Probit model in SPSS 27.0 (SPSS Inc., Chicago, IL, USA) to calculate the LC_50_ and 95% confidence interval. Using the LC_50_ of the susceptible strain as the reference, the relative resistance ratio of different field populations to each insecticide was calculated separately, with the following formula: Resistance ratio (RR) = LC_50_ of the tested population/LC_50_ of the susceptible strain. Based on the classification standard established by the China Pesticide Industry Association, pest resistance levels were categorized as follows: low resistance (5.00 < RR ≤ 10.00) medium resistance (10.00 < RR ≤ 100.00), and high resistance (RR > 100.00) [30].

## 3. Results

### 3.1. Monitoring of Insecticide Resistance in A. gossypii

In 2024 and 2025, insecticide resistance monitoring was conducted on field populations of *A. gossypii* from Hutubi, Manas, Shihezi, Shawan, Wusu, Jinghe, Bole, and Kuitun in Xinjiang. The study assessed resistance to sulfoxaflor, acetamiprid, imidacloprid, abamectin, and chlorpyrifos.

#### 3.1.1. Sulfoxaflor

The resistance levels of *A. gossypii* field populations to sulfoxaflor in 2024 and 2025 are shown in Table 1. In 2024, the resistance ratios (RRs) of the seven populations ranged from 2.19 to 6.85, indicating low resistance across all populations. The Jinghe population exhibited the highest resistance (RR = 6.85), whereas the Manas population showed the lowest (RR = 2.19). Compared with 2024, resistance to sulfoxaflor had increased in 2025, all seven populations, with RR values ranging from 10.55 to 21.49, reaching medium resistance levels. The Shihezi and Manas populations displayed the highest resistance (RR = 21.49 and 21.44, respectively), while the Wusu population had comparatively lower resistance (RR = 10.55).

#### 3.1.2. Acetamiprid

The resistance levels of *A. gossypii* field populations to acetamiprid in 2024 and 2025 are presented in Table 2. In 2024, all six populations except Jinghe showed moderate resistance to acetamiprid, with RR values ranging from 35.99 to 66.67, while the Jinghe population showed low resistance (RR = 8.79). The Shihezi population displayed the highest resistance (RR = 66.67), whereas the Shawan population exhibited the lowest resistance (RR = 35.99). In 2025, the resistance levels of all seven field populations to acetamiprid declined, with RR values ranging from 15.46 to 43.20; however, all populations remained moderately resistant. Among these, the Jinghe population retained the highest resistance (RR = 43.20), and the Shawan population maintained the lowest resistance (RR = 15.46).

#### 3.1.3. Imidacloprid

The resistance levels of *A. gossypii* field populations to imidacloprid in 2024 and 2025 are shown in Table 3. In 2024, the resistance ratios of the seven field populations to imidacloprid ranged from 20.50 to 237.66. Among these, the Shihezi, Jinghe, and Manas populations reached high resistance, while the Shawan, Bole, Hutubi, and Wusu populations reached moderate resistance. In 2025, the resistance levels of the seven field populations to imidacloprid were similar to those in 2024, with resistance ratios ranging from 26.52 to 160.43. The Jinghe and Shawan populations showed high resistance, whereas the Shawan, Shihezi, Manas, Hutubi, and Wusu populations reached moderate resistance.

#### 3.1.4. Abamectin

The resistance levels of *A. gossypii* field populations to abamectin in 2024 and 2025 are shown in Table 4. In 2024, the resistance ratios of the seven field populations to abamectin ranged from 2.74 to 23.46. Among these, the Bole population was susceptible (RR = 2.74), while the Shawan and Jinghe populations exhibited low resistance (RR = 6.21 and 7.69, respectively). The Hutubi, Manas, Shihezi, and Wusu populations demonstrated moderate resistance (RR = 10.55–23.46). In 2025, the resistance ratios of the seven field populations to abamectin ranged from 4.34 to 14.41. The Shawan population was susceptible (RR = 4.34), whereas the Hutubi, Shihezi, Jinghe, and Kuitun populations displayed low resistance (RR = 5.58–7.58). The Manas and Wusu populations displayed moderate resistance (RR = 11.70 and 14.41, respectively).

#### 3.1.5. Chlorpyrifos

The resistance levels of *A. gossypii* field populations to chlorpyrifos in 2024 and 2025 are shown in Table 5. In both years, the RR values of all field populations to chlorpyrifos were below 5.00, indicating that all populations were susceptible to chlorpyrifos.

**Figure 1:**
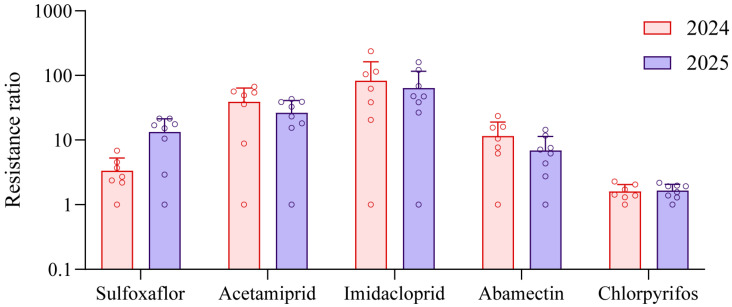
Average resistance ratios of *A. gossypii* field populations in 2024 and 2025.

Overall, among the eight *A. gossypii* field populations monitored over the two-year period, resistance to imidacloprid was the highest, followed by acetamiprid. Resistance to abamectin remained at relatively low levels in both years. In contrast, resistance to sulfoxaflor increased markedly from 2024 to 2025, rising from low to moderate levels in all populations. All populations remained consistently susceptible to chlorpyrifos (Figure 1).

### 3.2. Detection of Resistance Gene Frequency

#### 3.2.1. Acetylcholinesterase

The mutation frequencies of five sites in the AChE gene across eight field populations in 2024 and 2025 are summarized in Table 6. The S431F mutation was nearly fixed in all populations, with frequencies exceeding 99.60% in both years, indicating that this resistant genotype has become predominant under long-term selection pressure from carbamate and organophosphate insecticides in Xinjiang. In contrast, the F139L mutation remained consistently rare (<0.40%) across all populations and years, suggesting it may confer a fitness cost or is not under strong selection. The V332A mutation showed moderate and relatively stable frequencies, averaging approximately 33% in both years, though with considerable inter-population variation (range: 12.81–68.71% in 2024; 13.38–48.18% in 2025). The A302S and G221A mutations exhibited low but increasing frequencies from 2024 to 2025 (A302S: from 0.03–32.66% to 0.60–29.47%; G221A: from 0.00–10.20% to 0.00–15.50%), suggesting a potential risk of gradual spread in field populations. Notably, the distribution of these mutations varied substantially among populations and years, with Shihezi showing the highest A302S frequency in 2024 (32.66%) and Shawan showing the highest in 2025 (29.47%), while G221A was most frequent in Wusu in 2024 (10.20%) and in Shawan in 2025 (15.50%).

**Figure 2:**
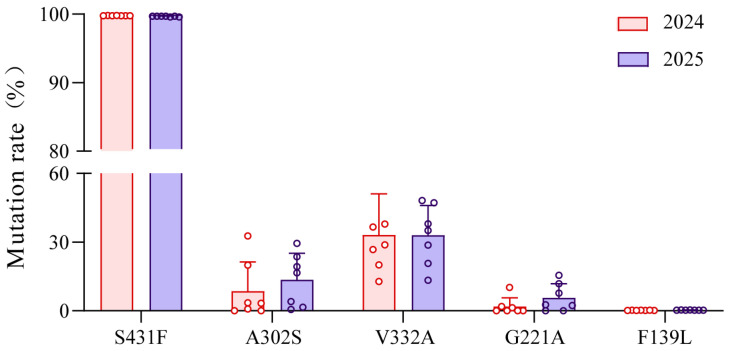
Average mutation rate of AChE gene in 2024 and 2025.

The comparison of mean mutation rates for five mutation sites in the AChE gene of *A. gossypii* field populations from 2024 to 2025 is presented in Figure 2. Among the five mutation sites, the S431F site exhibited the highest mutation rate, with mean mutation rate approaching 100% in both years (99.78% in 2024 and 99.67% in 2025), indicating that nearly all collected field populations of *A. gossypii* carried this mutation. The V332A site demonstrated relatively stable mutation rates, with mean values of approximately 30% over the two-year period (33.11% in 2024 and 33.02% in 2025). The mutation rates of A302S and G221A showed a slight increase during the two-year period (A302S: 8.61% in 2024 and 13.58% in 2025; G221A: 1.93% in 2024 and 5.72% in 2025). In contrast, the F139L site demonstrated a comparatively low mutation rate, with mean mutation rate of approximately 0.00% in both years (0.22% in 2024 and 0.30% in 2025).

#### 3.2.2. Nicotinic Acetylcholine Receptor *β*1 Subunit

The mutation frequencies of three sites in the nAChR *β*1 subunit across eight field populations in 2024 and 2025 are summarized in Table 7. From 2024 to 2025, the mutation rates of R81T and V62I in the *β*1 subunit of the nicotinic acetylcholine receptor (nAChR) in different field populations of *A. gossypii* remained approximately 50.00%. In contrast, the mutation rate of K264E was significantly lower, with all values below 1.00%.

**Figure 3:**
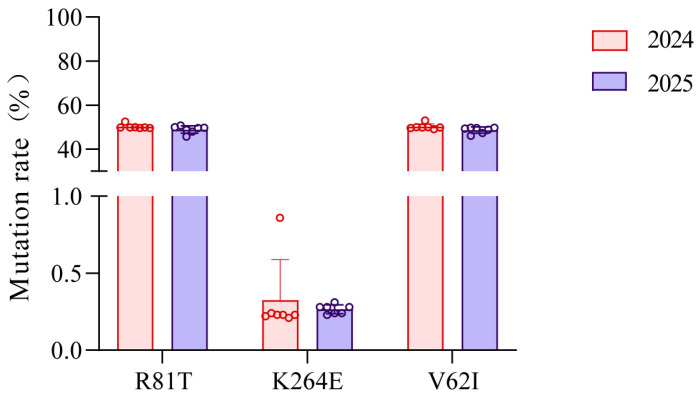
Average mutation rate in *β*1 subunit of nAChR in 2024 and 2025.

The following comparison of mutation rates at three mutation sites in the *β*1 subunit of the nAChR of *A. gossypii* field populations from 2024 to 2025 is demonstrated in Figure 3. Among these sites, R81T and V62I exhibited relatively stable mutation rates, maintaining consistent averages of approximately 50% across both years (R81T: 50.19% in 2024 and 48.91% in 2025; V62I: 50.21% in 2024 and 48.66% in 2025). In contrast, K264E showed significantly lower mutation rates, averaging approximately 0.00% in both years (0.33% in 2024 and 0.27% in 2025).

## 4. Discussion

In the 1990s, neonicotinoid insecticides (e.g., imidacloprid, thiamethoxam, dinotefuran, clothianidin, acetamiprid, and cycloxaprid) emerged as the predominant agents for controlling *A. gossypii* [31,32]. However, due to their large-scale and prolonged consecutive use, field populations of *A. gossypii* in provinces such as Shandong, Hebei, and Xinjiang have developed increasingly severe resistance to these insecticides [11,12,33,34].

This study systematically monitored changes in resistance levels of *A. gossypii* field populations to sulfoxaflor, acetamiprid, imidacloprid, abamectin, and chlorpyrifos in the northern Xinjiang cotton region from 2024 to 2025, providing a critical basis for the scientific selection of field insecticides and resistance management. Previous field monitoring has shown that *A. gossypii* populations in multiple cotton-growing regions of China have developed high resistance to imidacloprid [8,12]. In the present study, the eight monitored field populations exhibited the highest resistance to neonicotinoid insecticides, all reaching at least moderate resistance levels. Among them, three populations (Shihezi, Jinghe, and Manas) reached high resistance in 2024, while two populations (Jinghe and Shawan) reached high resistance in 2025. Consequently, imidacloprid is no longer suitable as a core insecticide for *A. gossypii* control. Sulfoxaflor, a sulfoximine insecticide, demonstrated low resistance in all monitored populations in 2024 (RR = 2.19–6.85), but resistance increased to moderate levels across all populations in 2025 (RR = 10.55–21.49), indicating that *A. gossypii* resistance to sulfoxaflor is continuously rising. Multiple studies have confirmed this increasing trend [12,13]. This rapid shift in resistance over a short period is likely closely related to the continuous and high frequency application of sulfoxaflor in local cotton fields. Chlorpyrifos remained susceptible (RR < 5.00) in all populations monitored over the two-year period, indicating extremely low minimal selection pressure in local cotton fields. Based on the monitoring results, the use of imidacloprid should be strictly limited for *A. gossypii* control in the Xinjiang cotton region. Sulfoxaflor and acetamiprid should be used in intra-season rotation or annual alternation. Chlorpyrifos can serve as a core rotational component in regions where *A. gossypii* populations exhibit high resistance to neonicotinoid insecticides.

Previous studies have identified mutations in the nAChR *β*1 subunit in field populations of *A. gossypii* across multiple provinces in China. In this study, we detected three mutations—R81T, V62I, and K264E—in populations collected from eight locations in northern Xinjiang. These mutations differ substantially in their functional roles and contributions to resistance. It is worth noting that V62I and K264E have not been functionally validated. They frequently co-segregate with R81T, suggesting they may represent linked haplotypes rather than independently functional resistance mutations. Nevertheless, monitoring these sites provides useful information for tracking the spread of resistant genotypes.

R81T is the only mutation among the three that has been functionally validated as a causal mutation conferring neonicotinoid resistance [35,36]. Located in the loop D region of the nAChR *β*1 subunit—a key component of the neonicotinoid binding site [26,37]—this substitution has been shown to reduce the binding affinity of neonicotinoids to the receptor [36,38]. Introduction of the homologous mutation in *Drosophila melanogaster* via CRISPR/Cas9 editing confirmed resistance to multiple neonicotinoids [39]. In our study, R81T was present at approximately 50% frequency across all populations in both years, likely contributing significantly to the moderate-to-high resistance observed for imidacloprid and acetamiprid. Unlike R81T, V62I has not been functionally validated. However, multiple lines of evidence support its utility as a molecular marker. First, its frequency was nearly identical to that of R81T across all populations and years, suggesting co-inheritance and representation of a distinct resistant haplotype. Second, both V62I and R81T were detected together in a highly imidacloprid-resistant strain [35]. Thus, while V62I may not itself confer resistance, its presence reliably indicates the R81T-bearing resistant haplotype. K264E was detected at extremely low frequencies in all populations. Although reported in some resistant populations [35], its functional significance remains unclear. Its consistently low frequency suggests a possible fitness cost, making it unlikely to contribute substantially to neonicotinoid resistance in Xinjiang populations.

Among the AChE mutations, S431F was predominant, with frequencies exceeding 99% in all populations across both years. This near-fixation reflects strong historical selection pressure from organophosphate and carbamate insecticides in Xinjiang [40,41], consistent with reports from central China [8] as well as observations in *Myzus persicae* [40] and *Sitobion miscanthi* [42]. However, S431F does not confer equivalent resistance to all carbamates and organophosphates. Functional studies indicate that S431F is primarily associated with high-level resistance to the carbamate pirimicarb [43], while its contribution to organophosphate resistance is variable and compound-dependent [44,45]. Recombinant AChE1 carrying S431F was insensitive to pirimicarb and omethoate but remained sensitive to demeton-S-methyl and exhibited hypersensitivity to carbofuran [44]. This compound-specificity explains why populations with near-fixed S431F can remain susceptible to certain organophosphates. The mutation rates of V332A, A302S, and G221A varied by year and population. V332A remained relatively stable, averaging approximately 33% in both years. A302S and G221A, though low in frequency, showed increasing trends from 2024 to 2025, suggesting a potential risk of gradual spread. Unlike S431F, A302S is associated with broader organophosphate resistance [46]. Clones carrying both A302S and S431F exhibited moderate resistance to profenofos and monocrotophos, whereas those with S431F alone did not [45], indicating that A302S contributes to organophosphate resistance beyond the effect of S431F. In contrast, F139L remained below 1% in all populations, suggesting either a fitness cost or lack of strong selection pressure. Previous studies have demonstrated that AChE mutations can confer resistance to organophosphates and carbamates [47,48]. In this study, despite the presence of multiple AChE mutations, all populations exhibited only low resistance to chlorpyrifos. This does not necessarily imply that these mutations have no effect on chlorpyrifos sensitivity—functional studies confirm that AChE mutations, particularly A302S, contribute to broad-spectrum organophosphate resistance [45,46], with compound-specific effects [44]. The observed susceptibility to chlorpyrifos may result from the varying impact of the same mutation on different organophosphates and the relatively low frequency of A302S, which may be insufficient to drive population-level resistance. For example, F290V has been shown to synergize with A302S in conferring chlorpyrifos resistance in other insects [49]. These findings are partially consistent with those of Shi et al. [8], but based on molecular evidence, minor contributions from these mutations cannot be excluded. Future studies using recombinant expression or molecular docking are needed to clarify their specific effects on chlorpyrifos binding.

This study has several limitations. Due to sample preservation constraints, genotyping of the susceptible strain was not conducted. An ideal experimental design would include a susceptible strain control to establish a more direct baseline. However, published data indicate that the mutations monitored in this study are either absent or extremely rare in susceptible laboratory strains and unselected field populations [8,35]. For instance, Shi et al. (2023) detected no S431F mutations in their susceptible strain [8], and Chen et al. (2017) found no R81T mutations in their susceptible colony [35]. Consequently, despite the absence of a direct control, the absolute mutation frequencies reported herein reliably represent deviations from the susceptible state. Future studies should incorporate parallel genotyping of susceptible strains to enable more rigorous comparisons.

## 5. Conclusions

Field populations of *A. gossypii* in the northern Xinjiang cotton-growing region have developed varying degrees of resistance to multiple insecticides, including neonicotinoids (acetamiprid and imidacloprid) and organophosphates (chlorpyrifos). All field populations examined carried multiple mutations in AChE (A302S, V332A, S431F, F139L, G221A) and the nAChR *β*1 subunit (R81T, K264E, V62I), with the high mutation frequencies of R81T and V62I in the nAChR *β*1 subunit likely being the main factors contributing to the high resistance observed against acetamiprid and imidacloprid. However, resistance development is a complex and dynamic process influenced not only by target-site mutations but also by multiple interacting ecological and evolutionary factors, including the availability of refugia for susceptible populations, the strength and continuity of selection pressure from insecticide applications, the fitness costs associated with resistance alleles, and the extent of gene flow between populations. Therefore, effective and sustainable management of resistance in *A. gossypii* requires a holistic and integrated approach that combines routine monitoring of resistance levels to all available chemistries, judicious use of new and existing insecticides with rotation between different mode of action groups to reduce selection pressure, preservation of susceptible populations through maintenance of refugia and non-chemical control tactics, and integration of molecular data with phenotypic bioassay results to enable early detection of emerging resistance. Together, these practices will provide IPM specialists with the necessary information to design evidence-based control strategies that combine and rotate effective chemistries to delay resistance development and prolong the efficacy of available insecticides. The findings of this study contribute to the growing resistance monitoring database for *A. gossypii* in Xinjiang and provide a scientific basis for the sustainable management of this key pest.

## Figures and Tables

**Table 1:** Toxicity of sulfoxaflor to *A. gossypii* field populations in 2024 and 2025.

Year	Population	Slope ± SE ^a^	LC_50_ mg/L (95%CL) ^b^	*ꭓ* ^2^	df	Resistance Ratio ^c^
2024	Sus strain	0.67 ± 0.08	7.16 (4.53–11.09)	9.04	3	1.00
	Manas	1.14 ± 0.11	15.67 (11.99–20.59)	7.81	3	2.19
	Wusu	1.21 ± 0.12	16.99 (13.17–22.08)	4.56	3	2.37
	Shihezi	0.94 ± 0.11	32.66 (23.57–48.50)	11.76	3	4.56
	Jinghe	1.20 ± 0.15	49.04 (37.71–67.73)	7.42	3	6.85
	Hutubi	1.10 ± 0.11	19.38 (14.74–25.95)	10.25	3	2.71
	Shawan	0.97 ± 0.11	26.59 (19.52–37.96)	4.29	3	3.71
	Bole	1.02 ± 0.11	20.80 (15.54–28.56)	2.48	3	2.91
2025	Sus strain	0.95 ± 0.11	5.01 (3.29–6.98)	6.36	3	1.00
	Manas	1.32 ± 0.16	107.42 (85.57–135.33)	4.13	3	21.44
	Wusu	1.23 ± 0.16	52.88 (38.21–67.92)	11.20	3	10.55
	Shihezi	1.03 ± 0.16	107.66 (80.71–144.85)	17.47	3	21.49
	Jinghe	1.36 ± 0.16	84.58 (66.90–105.05)	4.25	3	16.88
	Hutubi	1.40 ± 0.16	88.01 (69.80–108.81)	4.14	3	17.57
	Shawan	1.40 ± 0.16	76.27 (60.28–94.17)	9.49	3	15.22
	Kuitun	1.55 ± 0.17	58.23 (45.85–71.14)	3.62	3	11.62

^a^ Standard error. ^b^ Confidence limits. ^c^ Resistance ratio = LC_50_ of the tested population/LC_50_ of the susceptible strain. The same applies to the tables below.

**Table 2:** Toxicity of acetamiprid to *A. gossypii* field populations in 2024 and 2025.

Year	Population	Slope ± SE ^a^	LC_50_ mg/L (95%CL) ^b^	*ꭓ* ^2^	df	Resistance Ratio ^c^
2024	Sus strain	0.91 ± 0.08	5.05 (3.49–7.11)	10.88	3	1.00
	Manas	1.42 ± 0.16	274.97 (223.28–346.47)	11.84	3	54.45
	Wusu	1.54 ± 0.17	284.11 (233.94–352.32)	3.88	3	56.26
	Shihezi	1.21 ± 0.16	336.66 (263.46–455.37)	8.57	3	66.67
	Jinghe	1.69 ± 0.17	244.39 (204.17–294.65)	8.79	3	8.79
	Hutubi	1.07 ± 0.15	248.35 (189.81–334.33)	4.69	3	49.18
	Shawan	1.07 ± 0.15	181.77 (136.73–237.76)	8.68	3	35.99
	Bole	1.27 ± 0.16	195.20 (154.20–245.89)	9.15	3	38.65
2025	Sus strain	1.06 ± 0.15	6.61 (3.94–9.32)	7.80	3	1.00
	Manas	1.70 ± 0.18	119.37 (96.26–143.54)	1.84	3	18.06
	Wusu	1.59 ± 0.17	216.40 (178.22–262.95)	7.91	3	32.74
	Shihezi	1.84 ± 0.18	153.27 (127.62–181.42)	11.65	3	23.19
	Jinghe	1.95 ± 0.19	285.53 (242.09–340.06)	5.98	3	43.20
	Hutubi	1.63 ± 0.17	258.50 (214.25–315.46)	10.26	3	39.11
	Shawan	1.41 ± 0.16	102.19 (76.83–128.02)	16.48	3	15.46
	Kuitun	1.53 ± 0.17	124.81 (98.61–152.46)	9.72	3	18.88

**Table 3:** Toxicity of imidacloprid to *A. gossypii* field populations in 2024 and 2025.

Year	Population	Slope ± SE ^a^	LC_50_ mg/L (95%CL) ^b^	*ꭓ* ^2^	df	Resistance Ratio ^c^
2024	Sus strain	1.00 ± 0.09	7.82 (4.81–12.34)	11.62	3	1.00
	Manas	1.02 ± 0.17	817.98 (552.28–1591.71)	7.58	3	104.60
	Wusu	1.12 ± 0.16	160.28 (104.96–215.10)	8.26	3	20.50
	Shihezi	0.61 ± 0.15	1858.53 (998.46–8600.67)	11.16	3	237.66
	Jinghe	1.12 ± 0.16	897.45 (676.67–1330.59)	9.75	3	114.76
	Hutubi	1.25 ± 0.16	301.61 (232.25–379.93)	7.20	3	38.57
	Shawan	1.17 ± 0.16	488.63 (381.69–638.92)	4.23	3	62.48
	Bole	0.85 ± 0.15	369.01 (262.72–599.28)	15.80	3	47.19
2025	Sus strain	1.28 ± 0.13	5.36 (3.98–6.91)	8.03	3	1.00
	Manas	1.20 ± 0.16	255.22 (189.13–326.07)	5.76	3	47.62
	Wusu	1.21 ± 0.16	142.15 (93.49–190.01)	6.16	3	26.52
	Shihezi	1.14 ± 0.16	205.62 (143.62–268.93)	3.59	3	38.36
	Jinghe	0.97 ± 0.16	859.91 (624.62–1367.42)	10.69	3	160.43
	Hutubi	0.92 ± 0.15	367.17 (261.26–504.36)	5.58	3	68.50
	Shawan	0.94 ± 0.15	650.09 (477.15–971.78)	7.47	3	121.29
	Kuitun	0.84 ± 0.15	453.12 (320.26–657.74)	3.81	3	84.54

**Table 4:** Toxicity of abamectin to *A. gossypii* field populations in 2024 and 2025.

Year	Population	Slope ± SE ^a^	LC_50_ mg/L (95%CL) ^b^	*ꭓ* ^2^	df	Resistance Ratio ^c^
2024	Sus strain	1.23 ± 0.13	6.01 (4.08–8.99)	23.02	3	1.00
	Manas	1.91 ± 0.19	93.74 (75.89–111.71)	12.92	3	15.60
	Wusu	1.49 ± 0.16	141.02 (112.60–171.97)	8.65	3	23.46
	Shihezi	1.54 ± 0.17	96.46 (65.92–127.52)	19.95	3	16.05
	Jinghe	1.57 ± 0.17	46.21 (35.54–56.91)	17.03	3	7.69
	Hutubi	1.22 ± 0.16	63.43 (47.37–80.62)	8.63	3	10.55
	Shawan	1.94 ± 0.20	37.34 (29.37–45.10)	10.63	3	6.21
	Bole	0.96 ± 0.12	16.45 (9.09–24.68)	11.66	3	2.74
2025	Sus strain	1.65 ± 0.17	3.60 (2.73–4.47)	7.40	3	1.00
	Manas	1.58 ± 0.16	51.89 (42.59–63.09)	3.51	3	14.41
	Wusu	1.43 ± 0.16	42.13 (33.67–51.98)	7.15	3	11.70
	Shihezi	1.58 ± 0.16	25.68 (20.13–31.45)	16.50	3	7.13
	Jinghe	2.44 ± 0.22	27.30 (23.23–31.55)	11.20	3	7.58
	Hutubi	1.75 ± 0.18	22.24 (17.62–26.97)	13.16	3	6.18
	Shawan	1.80 ± 0.19	15.61 (11.88–19.27)	11.32	3	4.34
	Kuitun	2.37 ± 0.22	20.10 (16.74–23.51)	4.47	3	5.58

**Table 5:** Toxicity of chlorpyrifos to *A. gossypii* field populations in 2024 and 2025.

Year	Population	Slope ± SE ^a^	LC_50_ mg/L (95%CL) ^b^	*ꭓ* ^2^	df	Resistance Ratio ^c^
2024	Sus strain	1.67 ± 0.15	5.11 (3.43–7.24)	22.17	3	1.00
	Manas	1.60 ± 0.14	7.32 (5.91–8.97)	9.76	3	1.43
	Wusu	1.49 ± 0.13	8.77 (7.00–10.87)	12.46	3	1.72
	Shihezi	1.46 ± 0.13	6.64 (5.21–8.27)	15.97	3	1.30
	Jinghe	1.27 ± 0.12	11.72 (9.14–14.94)	9.02	3	2.29
	Hutubi	1.96 ± 0.17	7.11 (5.91–8.52)	11.76	3	1.39
	Shawan	1.83 ± 0.15	10.48 (8.64–12.67)	16.78	3	2.05
	Bole	1.47 ± 0.13	8.03 (6.38–9.99)	13.70	3	1.57
2025	Sus strain	2.54 ± 0.26	3.25 (2.68–3.80)	8.775	3	1.00
	Manas	2.02 ± 0.18	6.45 (5.38–7.61)	13.414	3	1.98
	Wusu	2.00 ± 0.18	6.27 (5.21–7.41)	11.531	3	1.93
	Shihezi	1.59 ± 0.16	4.51 (3.49–5.57)	17.226	3	1.39
	Jinghe	1.79 ± 0.17	7.08 (5.81–8.47)	15.119	3	2.18
	Hutubi	2.18 ± 0.19	6.26 (5.27–7.33)	15.813	3	1.93
	Shawan	2.34 ± 0.22	4.18 (3.48–4.89)	3.471	3	1.29
	Kuitun	1.74 ± 0.17	4.54 (3.60–5.52)	13.276	3	1.40

**Table 6:** Mutation rate of AChE gene in 2024 and 2025.

Gene	Year	Hutubi	Manas	Shihezi	Shawan	Wusu	Jinghe	Bole	Kuitun
A302S	2024	3.47	3.33	32.66	0.04	19.9	0.83	0.03	-
	2025	1.54	19.30	0.60	29.47	16.55	23.59	-	4.00
V332A	2024	12.81	26.78	68.71	28.86	36.64	37.87	20.10	-
	2025	35.04	38.00	20.69	47.15	28.73	48.18	-	13.38
S431F	2024	99.78	99.76	99.79	99.77	99.78	99.79	99.77	-
	2025	99.70	99.70	99.60	99.70	99.70	99.70	-	99.60
F139L	2024	0.22	0.19	0.24	0.21	0.21	0.24	0.20	-
	2025	0.34	0.27	0.38	0.25	0.26	0.32	-	0.31
G221A	2024	1.92	1.36	0	0	10.20	0	0	-
	2025	0.01	2.34	0	15.50	7.72	11.8	-	2.65

**Table 7:** Mutation rate of nAChR gene in 2024 and 2025.

Gene	Year	Hutubi	Manas	Shihezi	Shawan	Wusu	Jinghe	Bole	Kuitun
R81T	2024	49.99	52.51	49.83	49.86	49.60	49.91	49.62	-
	2025	50.00	48.70	49.70	45.70	50.70	48.00	-	49.60
K264E	2024	0.92	0.22	0.21	0.23	0.24	0.23	0.23	-
	2025	0.28	0.24	0.31	0.24	0.28	0.28	-	0.23
V62I	2024	49.90	53.00	49.90	49.60	50.00	50.00	49.10	-
	2025	49.60	48.80	49.30	46.10	49.80	47.30	-	49.70

## Data Availability

The original contributions presented in this study are included in the article/Supplementary Material. Further inquiries can be directed to the corresponding authors.

## Supplementary Materials

The following supporting information can be downloaded at https://www.mdpi.com/article/10.3390/insects17030314/s1. Table S1: Amplification primers specific to mutated loci of resistance-related genes.
