# Supplementary material for: Monitoring of Insecticide Resistance and Resistance-Related Point Mutations in Field-Collected Aphis gossypii Populations in the Northern Xinjiang, China
Source: Insects. 2026 Mar 13;17(3):314. doi: 10.3390/insects17030314 (PMC13027088; doi:10.3390/insects17030314)
Supplement: Supplementary file 1 [file insects-17-00314-s001.zip › insects-4146828-supplementary.pdf]

Table S1. Amplification primers specific to mutated loci of resistance-related genes

| Mutation site | Sequences |
|---|---|
| R81T-F | TAATACGATTTGTTGAAAAATAAATAGAATAAC |
| R81T-R | TCGGATAAGACGTCTAATACGCC |
| K264E-F | ATTACGTTCTACATAGTGATTCGAAGG |
| K264E-R | ATGAACGTGAATAGCAAGTATTTGGC |
| V62I-F | GTTGGTGTTCAGAAGACGAGGAG |
| V62I-R | ATCAATAATATTAAAATTAGTCACATATTGAACG |
| A302S-F/V332A-F | GATCAGCTAATGGCGTTGCAATG |
| A302S-R/V332A-R | CCCATTGCTTTTGCTAGTTTAAGTCC |
| S431F-F | CTATATGTTTCTTCCCGTTTGTTCCG |
| S431F-R | GCCGCATCTGCATTCGGATTAAG |
| G221A-F | TTCGCCGGTATCAGAAGATTGCC |
| G221A-R | TTCCTGGAACGTCTTCAGTGTCG |
| F139L-F | GGTGGGTCAGCTCAGGTTCC |
| F139L-R | CATGATGTGCGTTATTCGAATGGTG |
